# Evaluation of a Mixing versus a Cycling Strategy of Antibiotic Use in Critically-Ill Medical Patients: Impact on Acquisition of Resistant Microorganisms and Clinical Outcomes

**DOI:** 10.1371/journal.pone.0150274

**Published:** 2016-03-16

**Authors:** Nazaret Cobos-Trigueros, Mar Solé, Pedro Castro, Jorge Luis Torres, Mariano Rinaudo, Elisa De Lazzari, Laura Morata, Cristina Hernández, Sara Fernández, Alex Soriano, José María Nicolás, Josep Mensa, Jordi Vila, José Antonio Martínez

**Affiliations:** 1 Department of Infectious Diseases, Hospital Clínic, IDIBAPS, Barcelona University, Barcelona, Spain; 2 ISGlobal, Barcelona Center for International Health Research (CRESIB), Hospital Clínic - University of Barcelona, Barcelona, Spain; 3 Medical Intensive Care Unit, Hospital Clínic, IDIBAPS, Barcelona University, Barcelona, Spain; 4 Health Biostatistic, Hospital Clínic-IDIBAPS, University of Barcelona, Barcelona, Spain; 5 Department of Clinical Microbiology, Hospital Clinic, School of Medicine, University of Barcelona, Barcelona, Spain; Tel Aviv University, ISRAEL

## Abstract

**Objective:**

To compare the effect of two strategies of antibiotic use (mixing vs. cycling) on the acquisition of resistant microorganisms, infections and other clinical outcomes.

**Methods:**

Prospective cohort study in an 8-bed intensive care unit during 35- months in which a mixing-cycling policy of antipseudomonal beta-lactams (meropenem, ceftazidime/piperacillin-tazobactam) and fluoroquinolones was operative. Nasopharyngeal and rectal swabs and respiratory secretions were obtained within 48h of admission and thrice weekly thereafter. Target microorganisms included methicillin-resistant *S*. *aureus*, vancomycin-resistant enterococci, third-generation cephalosporin-resistant Enterobacteriaceae and non-fermenters.

**Results:**

A total of 409 (42%) patients were included in mixing and 560 (58%) in cycling. Exposure to ceftazidime/piperacillin-tazobactam and fluoroquinolones was significantly higher in mixing while exposure to meropenem was higher in cycling, although overall use of antipseudomonals was not significantly different (37.5/100 patient-days vs. 38.1/100 patient-days). There was a barely higher acquisition rate of microorganisms during mixing, but this difference lost its significance when the cases due to an exogenous *Burkholderia cepacia* outbreak were excluded (19.3% vs. 15.4%, OR 0.8, CI 0.5–1.1). Acquisition of *Pseudomonas aeruginosa* resistant to the intervention antibiotics or with multiple-drug resistance was similar. There were no significant differences between mixing and cycling in the proportion of patients acquiring any infection (16.6% vs. 14.5%, OR 0.9, CI 0.6–1.2), any infection due to target microorganisms (5.9% vs. 5.2%, OR 0.9, CI 0.5–1.5), length of stay (median 5 d for both groups) or mortality (13.9 vs. 14.3%, OR 1.03, CI 0.7–1.3).

**Conclusions:**

A cycling strategy of antibiotic use with a 6-week cycle duration is similar to mixing in terms of acquisition of resistant microorganisms, infections, length of stay and mortality.

## Introduction

Prevalence of resistance is causally linked to the prevalence of antibiotic use, hence curtailing the use of antibiotics will lower the rate of resistance and changes in response to restrictive policies are expected to occur over the span of weeks in the hospital setting [1–5]. In order to decrease the prevalence of use of any given antibiotic over a defined period of time, two non-mutually exclusive general approaches can be pursued: to diminish the number of indications and/or to shorten the duration of administration or to promote heterogeneity of use.

Several interventions directed to increase heterogeneity of antibiotic use have been envisaged. One of them, so-called cycling or rotation, consists of the sequential use of antibiotics not sharing a common mechanism of resistance. Alternatively, all available antibiotics can be used concurrently in different patients, a strategy so-called mixing [6–8]. Cycling promotes diversification of use when the whole period encompassed by the different cycles is considered, while mixing would theoretically provide heterogeneity in a constant manner. Other ways of achieving diversification are what has been denominated as “dual cycling” (meaning that in each period different antibiotics are given depending on the type of infection, hence some mixing of two drugs is guaranteed within the periods) [9,10] and the strategy named “periodic antibiotic monitoring and supervision (PAMS)” [11]. In PAMS, a set of several antibiotics is selected for intervention and each component is either promoted, restricted or left off-supervision during a scheduled period of time (usually three months) according to their frequency of use and/or the prevalence of resistance of an indicating microorganism (e.g. *P*. *aeruginosa*) observed during the preceding term.

Although there is enough evidence that homogeneous use of a single antibiotic class will lead to a rapid increase of resistance in ICUs, there is not a definitive answer to the question of which diversification strategy would be the best. In regards to cycling, there is still concern about the possibility that it could, under some conditions such as an excessive duration of the periods of predominant use of an antimicrobial class, actually increase resistance [9,12,13]. On the other hand, direct comparisons among different diversification strategies are scarce and only two prior studies have specifically addressed the issue of comparing cycling versus mixing [12,14] in the ICU setting with contradictory results.

The decision for implementing a policy of alternating mixing-cycling periods of antibiotic use on a regular basis in a medical intensive care unit in our center gave us the opportunity to evaluate the relative benefits of these two strategies of antibiotic diversification in terms of acquisition of resistant microorganisms, infections due to these and other clinical outcomes. The present study is an analysis of the data gathered during the first three years of implementation of such policy.

## Materials and Methods

### Study Population

From February 2006 to December 2008, all patients admitted to an eight-bed adult medical ICU of a 700-bed university hospital were prospectively included in the study. The study unit has two individual rooms and a central space with six cubicles, and it is the reference unit for critically ill medical patients from Internal Medicine, Hematology, Oncology and Infectious Diseases wards.

After a previous pilot experience [14], the director of the study unit decided to implement a mixing-cycling strategy of antibiotic use on a regular basis. In order to evaluate such policy, a prospective study of systematic screening for the detection of resistant or potentially resistant microorganisms was carried out during the first three years of implementation and a post-hoc analysis was performed. The study protocol was approved by the Research Ethics Committee of the Hospital Clinic of Barcelona, which waived the requirement of informed consent (approval reference number 2616).

### Microbiological procedures

Swabbing of nares, pharynx and rectum, and respiratory secretions (tracheobronchial aspirate, bronchoscopic samples or sputum) were obtained within 48 hours of admission and thrice weekly thereafter during the first two months of ICU stay or until being discharged or death, whatever happened first. Other clinical samples were obtained as deemed necessary by the attending physician. Samples were cultured in conventional and selective agar media for the isolation of extended spectrum beta-lactamase producing Enterobacteriaceae, non-fermentative Gram-negative bacilli, methicillin-resistant *Staphylococcus aureus* and vancomycin-resistant enterococci. No environmental cultures were taken. Susceptibility testing was done by a microdilution technique according to the CLSI guidelines [15]. For the purpose of analysis, intermediate susceptibility was considered as resistance.

### Clinical variables

Demographics, clinical variables, severity scores (APACHE II and SOFA) on admission and exposures during ICU stay were prospectively collected from all admitted patients as previously described [14].

### Antibiotic use

For the duration of the study, a policy of consecutive mixing-cycling periods of three classes of antipseudomonal agents (meropenem, ceftazidime/piperacillin-tazobactam and ciprofloxacin/levofloxacin) was implemented in the study unit. Each mixing period lasted 4.5 months. During mixing, a different antipseudomonal class was prescribed to each consecutive patient. Cycling periods were intended to be composed of three consecutive 6-week intervals in which a different antibiotic class was preferentially administered. However, due to administrative reasons, the unit was closed for one month after the first 6-week interval of two of the cycling periods. Therefore, the team decided to do an entire 4.5-month period of cycling after the unit was reopened. For the purpose of the analysis the 6-week pre-closing interval plus the 4.5-months after-reopening were considered as a whole cycling period. The order of the class of antibiotic was chosen at random at the beginning of each cycling period. Wash-out periods were not performed either between cycling intervals or mixing and cycling periods. The decision to provide antipseudomonal coverage was made by the attending physician based on clinical judgment. To administer combination treatment with a beta-lactam and a fluoroquinolone or aminoglycoside was also a decision of the attending physician and, based on current protocols, it was only encouraged for patients with severe sepsis. Amikacin in a once-daily dose was the aminoglycoside favored for antipseudomonal coverage combination, but its administration as monotherapy or for >5 days was discouraged.

### Definitions

**Resistant or potentially resistant microorganisms (RPRMs):** methicillin-resistant *Staphylococcus aureus* (MRSA), vancomycin-resistant enterococci (VRE), enteric Gram-negative bacilli resistant to third-generation cephalosporins (cefotaxime, ceftazidime or both), and non-fermentative Gram-negative bacilli (*Pseudomonas aeruginosa*, *Burkholderia cepacia*, *Stenotrophomonas maltophilia*, *Pseudomonas spp* and *Acinetobacter baumannii*). **Multidrug resistant (MDR) *P*. *aeruginosa*:** *P*. *aeruginosa* non-susceptible to at least one agent in three or more antimicrobial categories as described elsewhere [16]. **Colonization** was defined as the isolation of a RPRM from a surveillance culture or non-sterile clinical sample. Patients with a RPRM isolated within 48 hours of ICU admission were considered to be colonized on admission. Organisms isolated 48 hours after admission in patients with previous negative specimens were considered as ICU-acquired. **Infection** was considered the reason for admission when the organic failure leading to critical care was meant to be a direct consequence of either the dysfunction of the infected organ or sepsis. Septic shock was defined according to the SCCM/ESICM/ACCP/ATS/SIS consensus conference [17]. ICU-acquired sepsis was defined as sepsis occurring more than 48 hours after admission to the ICU. Catheter-related bacteremia was defined according to IDSA guidelines [18]. The diagnosis of pneumonia required the presence of new and/or progressive infiltrates in chest radiograph, and at least two of the following criteria: fever ≥38°C or hypothermia ≤35°C, leukocytosis ≥12000/μL or leucopenia <4000/μL, or purulent respiratory secretions. When the patient was invasively ventilated for more than 48 h, pneumonia was considered ventilator-associated pneumonia (VAP) [19]. Patients without radiological criteria of pneumonia but fulfilling the above-mentioned clinical criteria were considered to have tracheobronchitis. Other infections were diagnosed according to the CDC criteria [20]. **Exposure to antibiotics** meant at least 24 hours of treatment.

### Epidemiological variables

Results of surveillance cultures were communicated to the attending physician either when they yielded a microorganism requiring contact precautions according to current isolation practices in the hospital (MRSA,VRE, enteric gram-negative bacilli producing extended-spectrum beta-lactamases, *P*. *aeruginosa* resistant to at least three classes of antipseudomonal agents considering ceftazidime and piperacillin-tazobactam or ciprofloxacin and levofloxacin as single classes) or an outbreak was suspected. Contact precautions implied the transfer to an individual room when available and, in any case, the wearing of disposable gowns and gloves when entering the cubicle or room. Patients with prior MRSA, multiple resistant gram-negative bacilli and VRE were automatically identified by an electronic tag on admission. Preventive isolation based on risk factors was not performed. Hand hygiene was primarily based in alcohol-hand rubs. Decolonization with mupirocin was only carried out in patients with MRSA present exclusively in nares. Clorhexidine was used for oral hygiene but not for body bathing. Selective decontamination of the digestive tract or any additional practice such as the use of extraordinary prophylactic antibiotics (except as clinically recommended in neutropenic, cirrhotic or HIV patients) was not performed during the study. There were no changes in isolation or hand hygiene practices during the study period.

### Statistical Analysis

This was a post-hoc analysis of clinical practice data without any statistical analysis plan defined *a priori*. For the purposes of analysis, all patients included in the mixing periods were compared against all those in the cycling periods. For continuous variables, means (standard deviation, SD) were used as measures of central tendency (dispersion). Denominators in proportions were always “number of patients”. Antibiotic use was expressed as the percentage of patients exposed to a given antibiotic and also as the density incidence of antibiotic use (the number of days on a given antibiotic per 100 patient-days of ICU stay for the considered period). Proportions were compared by using the χ^2^ or Fisher’s exact test, if more than 20% of the expected counts were ≤5 or at least one individual expected count were <1. Continuous variables were compared by using the t-test or Mann-Whitney test in case of normality assumption violation. Acquisition of any RPRM, acquisition of any infection due to RPRM and acquisition of any infection were considered as outcome variables. In order to analyze the factors influencing each outcome, a random effects logistic regression model was first estimated defining individual observations as the lower level nested within the upper level defined as the mixing/cycling groups. The intraclass correlation coefficient was tested against zero using the likelihood ratio test and due to the fact that no statistical significance was reached for any of the outcomes, logistic regression with only individual level variation was used. Factors evaluated in the multiple regression were chosen using clinical judgment and statistical criteria (simple regression p-value<0.05). Predictors in the multiple final model were selected using a backward stepwise process. Analyses were done by using SPSS 20.0 version statistical package or Stata (StataCorp. 2013. Stata: Release 13. Statistical Software. College Station, TX: StataCorp LP).

## Results

### Patients’ characteristics and clinical events

During the 35-month study period, 969 patients were admitted to the unit, of which 409 (42%) were included in three mixing periods and 560 (58%) in three cycling periods. Patients’ characteristics at ICU admission during the corresponding mixing and cycling periods are shown in Table 1. Patients in the cycling periods had more frequently previous surgery, urinary tract infection, catheter related bacteremia, shock and a higher APACHE and SOFA scores. There were no differences in length of stay or mortality. Non-antibiotic exposures during ICU stay are shown in Table 2. More patients in the mixing periods received enteral nutrition while more patients during cycling underwent surgery.

**Table 1:** Patients’ characteristics on admission and outcomes in each period.

Characteristics	Mixing (n = 409)	Cycling (n = 560)	OR (CI 95%)	P
**Age ≥60 years old**	**240 (58.7)**	**298 (53.2)**	**0.8 (0.6–1)**	**0.09**
**Pre-ICU stay >3 days**	**85 (20.8)**	**135 (24.1)**	**1.2 (0.9–1.7)**	**0.2**
**Underlying diseases and other conditions**				
Solid organ cancer	49 (12)	51 (9.1)	0.74 (0.5–1.1)	0.2
Hematological malignancy	45 (11)	82 (14.6)	1.39 (0.9–2)	0.1
HSCT	15 (3.7)	31 (5.5)	1.54 (0.8–2.9)	0.2
Heart failure	24 (5.9)	44 (7.9)	1.37 (0.8–2.3)	0.2
Surgery prior ICU admission	76 (18.6)	143 (25.5)	1.5 (1.1–2.1)	0.01
Prior antibiotic (≤ 1 month)	112 (27.4)	172 (30.7)	1.18 (0.9–1.6)	0.3
Shock on admission	57 (13.9)	106 (18.9)	1.44 (1–2)	0.04
**Reason for admission**				
Respiratory disease	19 (4.6)	15 (2.7)	0.56 (0.3–1.1)	0.1
Postsurgical	33 (8.1)	66 (11.8)	1.52 (1–2.4)	0.06
**Prevalent infections on admission**				
Pneumonia	127 (31.1)	143 (25.5)	0.76 (0.6–1)	0.06
Primary bacteremia	6 (1.5)	13 (2.3)	1.6 (0.6–4.2)	0.3
Urinary tract infection	11 (2.7)	32 (5.7)	2.19 (1.1–4.4)	0.02
Catheter related bacteremia	1 (0.2)	11 (2)	8.2 (1.1–63.6)	0.02
**Microorganisms on admission**^a^				
*P*. *aeruginosa*	24 (5.9)	48 (8.6)	1.5 (0.9–2.5)	0.1
*B*. *cepacia*	1 (0.2)	1 (0.2)	0.7 (0–11.7)	0.8
*S*. *maltophilia*	2 (0.5)	4 (0.7)	1.5 (0.3–8)	0.7
*A*.*baumanii*	1 (0.2)	3 (0.5)	2.2 (0.2–21.2)	0.5
*Pseudomonas spp*	1 (0.2)	1 (0.2)	0.73 (0–11.7)	0.8
*Klebsiella* resistant to 3gCEF	2 (0.5)	9 (1.6)	3.3 (0.7–15.5)	0.1
*E*. *coli* resistant to 3gCEF	15 (3.7)	27 (4.8)	1.3 (0.7–2.5)	0.4
Other GNB resistant to 3gCEF	5 (1.2)	15 (2.7)	2.2 (0.8–6.2)	0.1
Methicillin-resistant *S*. *aureus*	17 (4.2)	14 (2.5)	0.6 (0.3–1.2)	0.2
Any RPRMs	61 (14.9)	107 (19.1)	1.4 (1–1.9)	0.1
**Severity scores**				
APACHE II score	18.9 (6.4)	20.1 (6.8)	-	0.006
SOFA score	5.9 (3.9)	6.7 (3.4)	-	0.001
**Outcomes**				
Length of ICU stay	5 (3–9)	5 (3–9)	-	0.2
In-ICU mortality	57 (13.9)	80 (14.3)	1.03 (0.7–1.5)	0.9
In hospital mortality	95 (23.2)	129 (23)	0.99 (0.7–1.3)	0.9

ICU, Intensive Care Unit; HSCT, haematopoietic stem cell transplantation. GNB, Gram-negative bacilli. RPRMs, resistant or potentially resistant microorganism. APACHE, Acute Physiology and Chronic Health Evaluation; SOFA, Sequential Organ Failure Assessment. 3gCEF, third generation cephalosporins. Categorical variables are expressed as number of patients (%) and continuous variables as mean (standard deviation). Other variables with a p-value >0.3 not shown include the following: male gender, neutropenia, solid organ transplantation, Human Immunodeficiency Virus infection, liver cirrhosis, chronic obstructive pulmonary disease, hemodialysis, diabetes, prior corticosteroids and immunosuppressive therapy, admission within the previous year and infection, cardiovascular, central nervous system and other diseases as reasons for admission.

^a^. Corresponds to the total number of acquired RPRMs ((colonization plus infection).

**Table 2:** Antibiotic and other exposures during ICU stay.

In-ICU exposures	Mixing (n = 409)	Cycling (n = 560)	OR (CI 95%)	P
**Non-antibiotic exposures**				
Intubation	247 (60.4)	307 (54.8)	0.8 (0.6–1)	0.1
Enteral nutrition	121 (29.6)	133 (23.8)	0.74 (0.6–1)	0.04
Tracheostomy	76 (18.6)	83 (14.8)	0.76 (0.5–1.1)	0.1
Surgery	25 (6.1)	64 (11.4)	1.98 (1.2–3.2)	<0.001
Blood transfusion	115 (28.1)	189 (33.8)	1.3 (1–1.7)	0.06
Corticosteroids	219 (53.5)	273 (48.8)	0.83 (0.6–1.1)	0.1
**Antibiotic exposures**				
Quinolone	159 (38.9)	157 (28)	0.61 (0.5–0.8)	<0.001
Meropenem	118 (28.9)	185 (33)	1.22 (0.9–1.6)	0.2
Ceftazidime	58 (14.2)	45 (8)	0.53 (0.4–0.8)	0.002
Piperacilline-tazobactam	82 (20)	106 (18.9)	0.93 (0.7–1.3)	0.7
Amikacin	16 (3.9)	38 (6.8)	1.79 (1–3.3)	0.05
Colistin	5 (1.2)	11 (2)	1.62 (0.6–4.7)	0.4
Other penicilins	111 (27.1)	115 (20.5)	0.69 (0.5–0.9)	0.02
Macrolide	3 (0.7)	30 (5.4)	7.66 (2.3–25.3)	<0.001
Linezolid	22 (5.4)	18 (3.2)	0.58 (0.3–1.1)	0.1
Any antibiotic	349 (85.3)	462 (82.5)	0.8 80.6–1.2)	0.2
Any antipseudomonal	263 (64.3)	350 (62.5)	0.93 (0.7–1.2)	0.6

ICU, Intensive Care Unit. Variables are expressed as number of patients (%). Other variables not included with p≥0.2 are central venous catheter, arterial catheter, bladder catheterization, parenteral nutrition, rectal tube, endoscopy, renal replacement therapy, other cephalosporins, glycopeptides, clindamycin, metronidazole, trimethoprim-sulfamethoxazole, fluconazole, and other antifungals.

### Antibiotic use

An antibiotic was prescribed to 811 (83.6%) patients of which 613 (63.3%) received an antipseudomonal agent. The proportion of patients receiving antibiotics is shown in Table 2 and the density incidence of use of the intervention antipseudomonals is shown in Table 3. During mixing, a higher proportion of patients received fluoroquinolones, ceftazidime and other penicillins (mostly amoxicillin-clavulanic acid), while during cycling, more patients received amikacin and a macrolide. However, in terms of incidence density of use, exposure to ceftazidime/piperacillin-tazobactam and fluoroquinolones was significantly higher during mixing while exposure to meropenem was higher during cycling. Although there was a slight increase in the density of use of any antipseudomonal during cycling (38.1/100 patient-days vs. 37.5/100 patient-days), the difference did not reach statistical significance (p = 0.1).

**Table 3:** Aggregated incidence density of antibiotic use in the different periods.

Periods (no. of patients)	Meropenem	Quinolones	Ceftazidime/Pip-taz
Mixing (409)^a^	24.7^b^	29.4^c^	25.5^d^
Cycling (560)	29	21.5	20.3
Meropenem intervals (196)	48.1^e^	16.7	12.5
Quinolone intervals (171)	23.1	41.5^e^	16.3
Ceftazidime/Pip-taz intervals (193)	15.6	11.3	30.5^e^

Pip-taz, piperacillin-tazobactam.

a: p≤0.001 compared with the incidence density of antibiotic use of the same antibiotic during cycling.

b vs. c: p<0.0001;

c vs. d: p = 0.001;

b vs. d: p = 0.6.

e: p<0.0001 compared with other antibiotics within the period and with the same antibiotic between different cycling intervals.

The density of use of the antibiotics under intervention throughout the different mixing and cycling periods is shown in S1 Fig. With some exceptions, antibiotics were used accordingly with the intended strategy (Table 3). During mixing periods, the density of use of meropenem and piperacillin-tazobactam was similar; however, quinolones were more heavily used than any of the other antipseudomonal classes. During cycling, as a whole, the scheduled agent was the most frequently and densely used within the corresponding periods and its use was higher than in the other intervals. During the corresponding cycle, meropenem, quinolones and ceftazidime/piperacillin-tazobactam were administered to 78% (range 73–82%), 76% (range 58–86%) and 65% (range 46–81%), of patients receiving an antipseudomonal agent, respectively. In addition, the incidence of use of the scheduled antibiotic was 1.8 to 2.8-fold higher than that of the other antipseudomonals. The only exception occurred in the last cycling period where ceftazidime/piperacillin-tazobactam did not increase during the scheduled interval (detailed density of antibiotic use in the different periods is shown in S1 Table).

### Microorganisms and infections acquired during ICU stay

During the study period, a total of 9961 surveillance samples were obtained and cultured, of which 1689 were from the lower respiratory tract, 2783 from the pharynx, 2809 from the nares and 2680 from the rectum. The mean number per included patient was 3.2 for nasal swabs, 3.1 for pharyngeal swabs, 3 for rectal swabs and 1.9 for respiratory samples (3.3 in intubated patients).

The distribution of microorganisms and infections acquired in the ICU during mixing and cycling periods is shown in Table 4. It is of note that during the first mixing period there was an outbreak of *B*. *cepacia* affecting six patients (2 colonized and 4 infected) due to moisturizing body milk contaminated during the manufacturing process. In regards to acquisition of RPRMs, there was a significant increase in the prevalence of acquisition of *B*. *cepacia* during mixing (entirely attributable to the above mentioned outbreak) and a non-significant trend towards more *S*. *maltophilia* during cycling (unrelated to any recognizable outbreak). In general, there was a significantly higher acquisition rate of RPRMs during mixing, but this difference lost its statistical significance when the excess cases due to the exogenous *B*. *cepacia* outbreak were not taken into account. In regard to infections, a significantly higher proportion of patients acquired pneumonia during mixing, although this excess rate was not due to RPRMs. As a whole, the proportion of patients acquiring any infection in the ICU was not significantly different between mixing and cycling.

**Table 4:** Microorganisms and infections acquired during ICU stay ^a^^,^^b^.

Microorganisms	Mixing (n = 409)	Cycling (n = 560)	OR (CI 95%)	P
*P*. *aeruginosa* acquisition	49 (12)	56 (10)	0.8 (0.5–1.2)	0.3
*P*. *aeruginosa* infection	17 (4.2)	23 (4.1)	1 (0.5–1.9)	1
*B*. *cepacia* acquisition^c^	7 (1.7)	1 (0.2)	0.1 (0–0.8)	0.01
*B*. *cepacia* infection^d^	4 (1)	1 (0.2)	0.2 (0–1.6)	0.1
*S*. *maltophilia* acquisition	1 (0.2)	9 (1.6)	6.7 (0.8–52.8)	0.051
*S*. *maltophilia* infection	0 (0)	6 (1.1)	0.6 (0.6–0.61)	0.04
*A*.*baumanii* acquisition	3 (0.7)	2 (0.4)	0.5 (0.1–2.9)	0.4
*A*.*baumanii* infection	0 (0)	1 (0.2)	0.6 (0.55–0.61)	0.4
*Pseudomonas spp* acquisition	2 (0.5)	2 (0.4)	0.7 (0.1–5.2)	0.8
*Pseudomonas spp* infection	0 (0)	1(0.2)	0.6 (0.55–0.6)	0.4
*Klebsiella* 3gCEF resistant acquisition	4 (1)	5 (0.9)	0.9 (0.2–3.4)	0.9
*Klebsiella* 3gCEF resistant infection	0	0	-	-
*E*. *coli* 3gCEF resistant acquisition	12 (2.9)	15 (2.7)	0.9 (0.4–2)	0.8
*E*. *coli* 3gCEF resistant infection	2 (0.5)	0 (0)	0.4 (0.39–0.45)	0.1
Other GNB resistant to 3gCEF acquisition	9 (2.2)	11 (2)	0.9 (0.4–2.2)	0.8
Other GNB resistant to 3gCEF infection	0 (0)	1 (0.2)	0.6 (0.55–0.6)	0.4
Methicillin-resistant *S*. *aureus* acquisition	8 (2)	7 (1.3)	0.6 (0.2–1.8)	0.4
Methicillin-resistant *S*. *aureus* infection	1 (0.2)	1 (0.2)	0.7 (0–11.7)	0.8
Any RPRMs acquisition ^e^	83 (20.3)	86 (15.4)	0.7 (0.5–1)	0.046
Any RPRMs infection^f^	24 (5.9)	29 (5.2)	0.9 (0.5–1.5)	0.6
**Infections**				
Tracheobronchitis	23 (5.6)	33 (5.9)	1.1 (0.6–1.8)	0.9
Tracheobronchitis due to RPRMs	13 (3.2)	14 (2.5)	0.8 (0.4–1.7)	0.5
Pneumonia^g^	29 (7.1)	22 (3.9)	0.5 (0.3–0.9)	0.03
Pneumonia due to RPRMs	13 (3.2)	13 (2.3)	0.7 (0.3–1.6)	0.4
Catheter-related bacteremia	17 (4.2)	21 (3.8)	0.9 (0.5–1.7)	0.8
Catheter-related bacteremia due to RPRMs	8 (2)	7 (1.2)	0.6 (0.2–1.8)	0.4
Primary bacteremia	12 (2.9)	16 (2.9)	1 (0.5–2.1)	0.9
Primary bacteremia due to RPRMs	4 (1)	7 (1.3)	1.3 (0.4–4.4)	0.7
Secondary bacteremia	11 (2.7)	8 (1.4)	0.5 (0.2–1.3)	0.2
Secondary bacteremia due to RPRMs	6 (1.5)	6 (1.1)	0.7 (0.2–2.3)	0.6
Urinary tract infection	5 (1.2)	5 (0.9)	0.7 (0.2–2.5)	0.6
Urinary tract infection due to RPRMs	3 (0.7)	3 (0.5)	0.7 (0.1–3.6)	0.7
Other infections	7 (1.7)	9 (1.6)	0.9 (0.3–2.5)	0.9
Other infections due to RPRMs	1 (0.2)	0 (0)	0.4 (0.39–0.45)	0.2
Any infection	68 (16.6)	81 (14.5)	0.9 (0.6–1.2)	0.4
Any infection due to RPRMs	24 (5.9)	29 (5.2)	0.9 (0.5–1.5)	0.6

ICU, Intensive Care Unit. RPRMs, resistant or potentially resistant microorganisms. 3gCEF, 3^rd^ generation cephalosporins. GNB, Gram-negative bacilli.

^a^. All figures except p-values are number of patients (%).

^b^. Acquisition corresponds to the total number of acquired microorganisms (colonization plus infection).

^c^. Excluding 6 cases from an exogenous outbreak of *B*. *cepacia*: mixing 1 (0.2%) and cycling 1 (0.2%) [OR 0.7 (0.1–11.7), *p =* 0.8].

^d^. Excluding 4 cases from a *B*. *cepacia* outbreak: mixing 0 (0) and cycling 1 (0.2%) [OR 0.6 (0.5–0.6), *p =* 0.4].

^e^. Excluding 4 cases from a *B*. *cepacia* outbreak: mixing 79 (19.3%) and cycling 86 (15.4%) [OR 0.8 (0.5–1.1), *p =* 0.1].

^f^. Excluding 4 cases from a *B*. *cepacia* outbreak: mixing 20 (4.9%) and cycling 29 (5.2%) [OR 1.1 (0.6–1.9), *p =* 0.8].

^g^.Of 51 acquired pneumonia, 44 were ventilator associated and 7 were non-ventilator associated [25 (6.1%) in mixing vs 19 (3.4%) in cycling, *p* = 0.04).

In multivariate analysis, cycling or mixing periods were not associated with the acquisition of any RPRM, infection due to RPRM or infection due to any microorganism in the ICU. Complete models are shown in S2 Table.

### *P*. *aeruginosa* resistance phenotypes in the different periods

The proportion of patients acquiring *P*. *aeruginosa* and its resistant phenotypes in the different study periods is shown in Table 5. There were no differences between mixing and cycling in regards to the prevalence of resistance to the different antipseudomonals or multiple drug resistance (MDR). During the intervals of preferential antibiotic use, there was not a statistically significant increase in the prevalence of resistance to the scheduled antibiotic compared with the resistance to that antibiotic during the periods of non-preferential use. However, during meropenem intervals, there was a trend towards an increase in resistance to carbapenems (p = 0.07), quinolones (p = 0.06), ceftazidime/piperacillin-tazobactam (p = 0.09) and MDR (p = 0.052) when compared with the ceftazidime/piperacillin-tazobactam intervals. A similar trend regarding quinolone resistance was also observed during the intervals of scheduled use of meropenem when compared with the mixing periods (p = 0.07).

**Table 5:** Number and proportion of patients acquiring *P*. *aeruginosa* and its resistance phenotypes in the different periods.

Periods (no. of patients)	All *P*.*aeruginosa*	Meropenem resistant	Quinolone resistant	Ceftazidime/Pip-taz resistant	Multidrugresistant
Mixing (409)	49 (12)	19 (4.6)	14 (3.4)^a^	18 (4.4)	12 (2.9)
Cycling (560)	56 (10)	27(4.8)	25 (4.5)	23 (4.1)	19 (3.4)
Meropenem intervals (196)	24 (12.2)	14 (7.1)^d^	13 (6.6)^a^^,^^c^	12 (6.1)^b^	10 (5.1)^e^
Quinolone intervals (171)	14 (8.2)	7 (4.1)	7 (4.1)	6 (3.5)	6 (3.5)
Ceftazidime/Pip-Taz intervals (193)	18 (9.3)	6 (3.1)^d^	5 (2.6)^c^	5 (2.6)^b^	3 (1.6)^e^

Pip-taz, Piperacillin-tazobactam. Comparisons between periods:

^a^. *p* = 0.07,

^b^. *p* = 0.09,

^c^. *p* = 0.058,

^d^. *p* = 0.07,

^e^. *p* = 0.052.

For all other comparisons: *p*>0.2.

## Discussion

The main finding of the present study is that mixing and cycling of antipseudomonal beta-lactams and fluoroquinolones with a cycle duration of 6 weeks are not significantly different in terms of overall rates of acquisition of RPRMs, ICU-acquired infections in general and those due to RPRMs, mortality and length of ICU stay.

Several prior studies have shown that for most antibiotics, cycling with a 3-month or longer intervals of predominant use can actually promote resistance in Gram-negative bacilli to the scheduled drug and even foster multiple-drug resistance or contribute to the development of outbreaks due to carbapenem-resistant *A*. *baumannii* or multiple-drug resistant *P*. *aeruginosa* [9,12,21–25]. Present data showed that in comparison with mixing, cycling was neither associated with acquisition of RPRMs nor with an increased prevalence in *P*. *aeruginosa* resistance phenotypes. In regards to *P*. *aeruginosa*, there was at most a non-significant trend during meropenem cycles towards a higher resistance to carbapenems, other intervention antibiotics and multiple-drug resistance than that observed during the periods of ceftazidime/piperacillin-tazobactam prioritization. This suggests that a 6-week span of predominant use of antipseudomonals can still be regarded as safe for preventing any significant increase in the prevalence of resistance of non-fermenters to these antibiotics, including carbapenems.

It is of note that an increase in the prevalence of resistance to a given antibiotic during its prioritization period is an obvious, time-dependent expectation, but not necessarily detrimental for keeping resistance under control over time. The key question is probably whether during the following cycles and before the reintroduction of a previously used drug, resistance will drop to levels equal or lower than those present at the onset of its last cycle of predominant use. Otherwise, a “ratchet effect” may result in a progressive increase in resistance with each reintroduction [26]. As a matter of fact, five prior studies in which cycles lasted less than 3 months [27–31] and two in which a 3-month rotation schedule was adopted [32,33] have shown sustained beneficial effects on Gram-negative bacilli resistance even for up to 6 years of continued application of the cycling strategy [30,31]. Furthermore, a recent meta-analysis [8] that included a very selected heterogeneous bundle of studies, some of which focused only on patients with febrile neutropenia [34,35], anti-Gram-positive agents [36] or aminoglycosides [37], concluded than in comparison with undefined baseline or homogenous use of antibiotics, cycling was associated with lower rates of infections in general, infections due to resistant pathogens and even a trend toward decreased mortality. It is of note that out of the six studies included in this meta-analysis which were carried out in ICUs and dealt with cycling of anti-Gram-negative antibiotics, the only study that proved to be ineffective and associated with an outbreak of resistant *P*. *aeruginosa* was the one in which the duration of cycles was longer (3 months) [23].

The relative merits of cycling versus mixing have been explored *in silico* [6–8,38] and in two previous interventional cohort studies [11,13]. Deterministic mathematical models consistently predict that mixing will always be superior to cycling in terms of reducing the evolution or the spread of resistance. However, some have suggested that cycling may perform better than mixing against multiple resistance [7,38] and recent models that assume a more real scenario in which empirical inactive antibiotics are expected to be changed by active ones, predict an advantage of cycling over mixing in most clinical situations whenever the duration of cycles is appropriate (around one month) [8]. Altogether, these constructions show that forecasting is highly dependent on fitting parameters and, therefore, outcomes from a similar strategy may be quite different depending on the epidemiological situation and practices prevailing in a particular clinical setting. Unfortunately, neither prior clinical studies have solved the uncertainty surrounding this issue. In the only study that specifically compared mixing with cycling [14], a single 4-month period of monthly rotation of antipseudomonal beta-lactams and ciprofloxacin was associated with a lower acquisition rate of *P*. *aeruginosa* resistant to cefepime when compared with a single 4-month mixing period. However, adherence to cycling was relatively poor with no more than 45% of patients receiving the scheduled antibiotic within a given cycle, meaning that there was, in fact, a good deal of mixing during the intended cycling. In the second work [12], several strategies producing different “rates” of mixing and cycling (4-month duration) were compared. It was observed that during the rotation period there was a lower Peterson index (i.e. less heterogeneity) than in the mixing periods and this was associated with an outbreak of a carbapenem-resistant *A*. *baumannii*, an increase in ESBL- producing *Enterobacteriaceae* and a higher incidence of *Enterococcus faecalis* infections.

The present study expands our previous experience [14] by making it possible to gather data throughout three consecutive alternating periods of mixing and cycling. Comparison of aggregated data indicates that performance of both strategies was quite similar. When looking at specific pathogens, more patients acquired *S*. *maltophilia* during cycling, but numbers were very low and the difference did not reach statistical significance. As a whole, the rate of acquisition of RPRMs was 4.9 percentage points higher in mixing than in cycling (p = 0.04), but this was due in part to a fortuitous *B*. *cepacia* outbreak caused by a contaminated skin-care product, obviously unrelated to the strategy. In regards to individual clinical infections, a higher proportion of patients acquired pneumonia during mixing but they were mostly not due to RPRMs, hence the relationship of this finding with that particular strategy seems unlikely.

The study has several caveats. It was performed in a single medical ICU of a university hospital, hence there may be some concern about the validity of the results in other settings. There was also an imperfect compliance with the strategies. In an ideal mixing scenario it would have been expected a similar incidence of use of the three intervention antibiotic classes, however, actually there was a higher, but not extraordinary, use of quinolones than beta-lactams. In regards to cycling periods, aggregate data showed that there was a significant increase in the prescription rate of the scheduled antibiotic with at least 65% of patients on antipseudomonals receiving the appropriate drug. However, an exception during the last cycling period occurred and ceftazidime/piperacillin-tazobactam use did not increase during the scheduled interval. On the other hand, exposure to ceftazidime/piperacillin-tazobactam and fluoroquinolones was significantly higher during mixing while exposure to meropenem was higher during cycling. Again, the differences were not dramatic, although a possible relationship of the increased use of meropenem during cycling with a higher frequency of *S*. *maltophilia* acquisition cannot be discarded. We think these limitations do not invalidate the interpretation of the general results and may be considered as part of the variability than can be expected in the real-world application of a general policy. Another potential confounder is the case-mix of patients in the different periods. Although the characteristics of patients were similar in many respects, those admitted during cycling periods had a higher prevalence of shock and significantly higher APACHE II and SOFA scores. However, the difference in shock was barely significant and the magnitude of the differences in the severity scores, though statistically significant, was of doubtful clinical meaning.

In conclusion, a cycling strategy of antibiotic use with a 6-week cycle duration is not significantly different to mixing in terms of acquisition of RPRMs, ICU-acquired infections in general and those due to RPRMs and other clinical outcomes such as length of ICU stay and mortality. In addition, a 6-week cycle may be considered as an appropriate safe time span for preventing any significant increase in the prevalence of *P*. *aeruginosa* resistance to beta-lactams and fluoroquinolones under conditions of predominant use. In order to provide antibiotic heterogeneity in the critical care setting, the choice between mixing and cycling should probably be based more on preferences or feasibility than in evidence of intrinsic differences in efficacy. Hopefully, a definitive answer to these issues may be obtained from an ongoing multicenter, cluster-randomized clinical trial [39].

## Supporting Information

S1 Fig Incidence density of antibiotic use in the different study periods. three mixing periods (M1, M2 and M3) alternating with three cycling periods. The first cycling was integrated by 3 intervals of predominant antibiotic use in the order ceftazidime/piperacillin-tazobactam (CT1) then quinolones (Q1) and finally meropenem (C1); the second one with 4 intervals in the order ceftazidime/piperacillin-tazobactam (CT2), then meropenem (C2) then quinolones (Q2) and finally ceftazidime/piperacillin-tazobactam (CT3); and the third one with 4 intervals in the order meropenem (C3) then quinolones (Q3) then meropenem (C4) and finally piperacillin-tazobactam (CT4). PIP-TAZ, piperacillin-tazobactam.
(TIF)

S1 Table Detailed incidence density of antibiotic use in the different periods. (DOCX)

S2 Table Multivariate analysis of factors associated with acquisition of any RPRM, infection due to RPRM and infection due to any microorganism in the ICU. (DOCX)
