# Supplementary figures and images for: Evaluation of a Mixing versus a Cycling Strategy of Antibiotic Use in Critically-Ill Medical Patients: Impact on Acquisition of Resistant Microorganisms and Clinical Outcomes
Source: PLoS One. 2016 Mar 16;11(3):e0150274. doi: 10.1371/journal.pone.0150274 (PMC4794237; doi:10.1371/journal.pone.0150274)

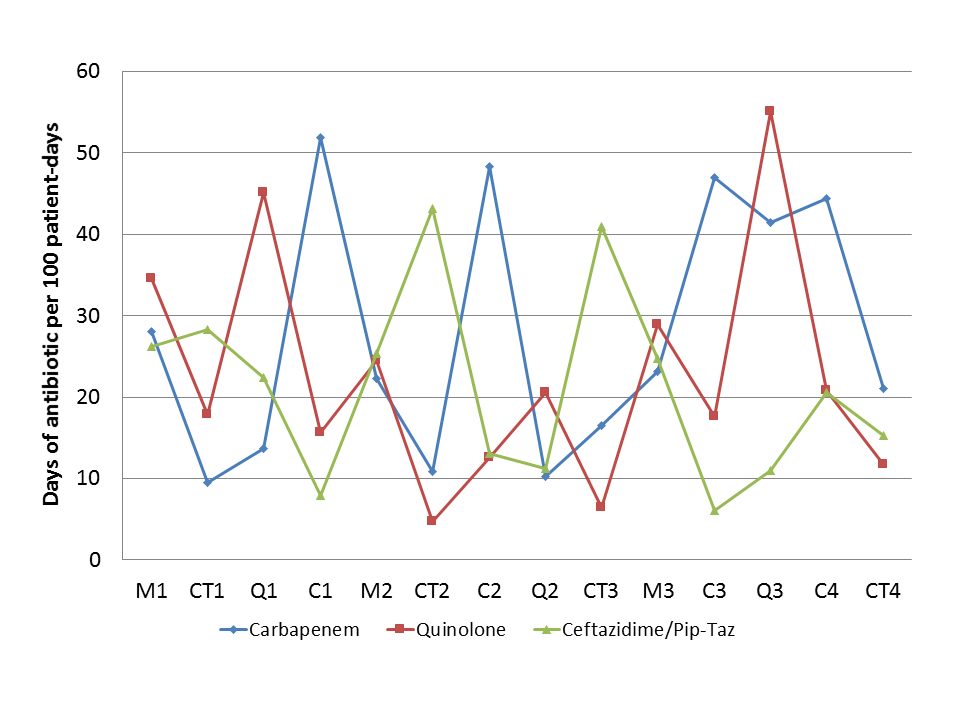

Supplement: S1 Fig — three mixing periods (M1, M2 and M3) alternating with three cycling periods. The first cycling was integrated by 3 intervals of predominant antibiotic use in the order ceftazidime/piperacillin-tazobactam (CT1) then quinolones (Q1) and finally meropenem (C1); the second one with 4 intervals in the order ceftazidime/piperacillin-tazobactam (CT2), then meropenem (C2) then quinolones (Q2) and finally ceftazidime/piperacillin-tazobactam (CT3); and the third one with 4 intervals in the order meropenem (C3) then quinolones (Q3) then meropenem (C4) and finally piperacillin-tazobactam (CT4). PIP-TAZ, piperacillin-tazobactam. (TIF) [file pone.0150274.s001.tif]
