# Supplementary material for: Evaluation of a Mixing versus a Cycling Strategy of Antibiotic Use in Critically-Ill Medical Patients: Impact on Acquisition of Resistant Microorganisms and Clinical Outcomes
Source: PLoS One. 2016 Mar 16;11(3):e0150274. doi: 10.1371/journal.pone.0150274 (PMC4794237; doi:10.1371/journal.pone.0150274)
Supplement: S1 Table — (DOCX) [file pone.0150274.s002.docx]

**Supplementary Table 1. Detailed incidence density of antibiotic use in the different periods.**

| **Period (no. of patients)** | **Meropenem** | **Quinolone** | **Ceftazidime/Pip-taz** |
| --- | --- | --- | --- |
| Mixing 1 (142) | 28 | 34.5 | 26.2 |
| CT1=ceftazidime/Pip-taz (52) | 9.5 | 17.9 | 28.3^a^ |
| Q1=quinolone (50) | 13.7 | 45.2^a^ | 22.3 |
| C1=meropenem (54) | 51.9^a^ | 15.7 | 7.9 |
| Mixing 2 (129) | 22.2 | 24.5 | 25.3 |
| CT2=ceftazidime/Pip-taz (43) | 10.9 | 4.8 | 43.2^a^ |
| C2=meropenem (46) | 48.3^a^ | 12.6 | 13 |
| Q2 =quinolone (61) | 10.3 | 20.5^a^ | 11.3 |
| CT3=ceftazidime/Pip-taz (42) | 16.5 | 6.5 | 41^a^ |
| Mixing 3 (138) | 23.2 | 28.9 | 24.7 |
| C3=meropenem (54) | 46.9^a^ | 17.6 | 6.1 |
| Q3 =quinolone (60) | 41.4 | 55.1^a^ | 10.9 |
| C4=meropenem (42) | 44.4^a^ | 20.8 | 20.6 |
| CT4=ceftazidime/Pip-taz (56) | 21.1 | 11.8 | 15.3^b^ |

**Pip-taz**, piperacillin-tazobactam. a. *p*<0.001 compared with the incidence density of use of the other antibiotics in the same period and with their own incidence density in the other intervals. b. *p>*0.2 compared with the incidence density of use of the other antibiotics in the same period.
