# Supplementary material for: Evaluation of a Mixing versus a Cycling Strategy of Antibiotic Use in Critically-Ill Medical Patients: Impact on Acquisition of Resistant Microorganisms and Clinical Outcomes
Source: PLoS One. 2016 Mar 16;11(3):e0150274. doi: 10.1371/journal.pone.0150274 (PMC4794237; doi:10.1371/journal.pone.0150274)
Supplement: S2 Table — (DOCX) [file pone.0150274.s003.docx]

Suplementary Table 2. Multivariate analysis of factors associated with acquisition of any RPRM, infection due to RPRM and infection due to any microorganism in the ICU.

| **Variable** | **OR (95% CI)** | **p** |
| --- | --- | --- |
| ***RPRM acquisition*** | | |
| Age ≥60 years old | 1.7 (1.2-2.5) | 0.008 |
| Emergency surgery prior ICU admission | 2.1 (1.2-3.6) | 0.009 |
| Admission due to non-septic respiratory disease | 3.3 (1.4-7.8) | 0.007 |
| Pneumonia on admission | 2.1 (1.3-3.2) | 0.001 |
| Primary bacteremia on admission | 4.5 (1.4-14.6) | 0.012 |
| Tracheostomy | 2 (1.2-3.3) | 0.012 |
| Intubation | 2.3 (1.4-3.8) | 0.001 |
| Parenteral nutrition | 2.5 (1.6-4.1) | <0.001 |
| Enteral nutrition | 2.9 (1.8-4.6) | <0.001 |
| Quinolones >3 days | 0.5 (0.3-0.8) | 0.008 |
| Other penicilins 1-3 days | 2.8 (1.6-4.8) | <0.001 |
| ***Infection due to RPRM*** | | |
| *P.aeruginosa* on admission | 3 (1.3-6.9) | 0.013 |
| Methicillin-resistant *S. aureus* on admission | 5.5 (1.6-19.4) | 0.008 |
| Tracheostomy | 3.6 (1.6-7.8) | 0.001 |
| Intubation | 5 (1.4-17.7) | 0.13 |
| Parenteral nutrition | 4.1 (2-8.4) | <0.001 |
| Enteral nutrition | 3.6 (1.7-7.6) | 0.001 |
| Corticoesteroids during the UCI stay | 2.4 (1.2-4.6) | 0.009 |
| Glycopeptides >3 days | 0.3 (0.1-0.7) | 0.004 |
| ***Any infection*** | | |
| Hematological malignancy | 3.1 (1.6-5.7) | 0.001 |
| Surgery prior ICU admission | 1.9 (1.2-3.1) | 0.01 |
| Tracheostomy | 3.3 (1.9-5.6) | <0.001 |
| Intubation | 3 (1.7-5.2) | <0.001 |
| Parenteral nutrition | 3.2 (1.9-5.3) | <0.001 |
| Enteral nutrition | 5.1 (3.1-8.3) | <0.001 |
| Glycopeptides >3 days | 0.4 (0.2-0.8) | 0.004 |

RPRM resistant or potentially resistant microorganisms. ICU intensive care unit.
